# 163例弥漫大B细胞淋巴瘤患者预后相关免疫表型研究

**DOI:** 10.3760/cma.j.issn.0253-2727.2021.06.008

**Published:** 2021-06

**Authors:** 鑫 杨, 舒 陈, 昱 祁, 晓莹 徐, 雪 管, 亦宸 杨, 岩雪 刘, 玉虹 郭, 文辰 巩, 亚男 高, 先火 王, 维 李, 兰芳 李, 凯 付, 会来 张, 斌 孟

**Affiliations:** 1 天津医科大学肿瘤医院，国家肿瘤临床医学研究中心，天津市肿瘤防治重点实验室，天津市恶性肿瘤临床医学研究中心，天津医科大学肿瘤医院中美淋巴血液肿瘤诊治中心 300060 Tianjin Medical University Cancer Institute and Hospital, National Clinical Research Center for Cancer; Key Laboratory of Cancer Prevention and Therapy, Tianjin; Tianjin's Clinical Research Center of Cancer, Tianjin 300060, China; 2 天津医科大学肿瘤医院病理科 300060 Department of Pathology, Tianjin Medical University Cancer Institute and Hospital, Tianjin 300060, China; 3 天津医科大学肿瘤医院淋巴瘤内科 300060 Department of Lymphoma, Tianjin Medical University Cancer Institute and Hospital, Tianjin 300060, China; 4 天津医科大学肿瘤医院中美淋巴血液肿瘤诊治中心，美国罗斯威尔帕克癌症研究所病理科 Department of Pathology, Roswell Park Cancer Institute, Buffalo, New York, USA

**Keywords:** 弥漫大B细胞淋巴瘤, 基因，BCL2, 基因，P53, 基因，BCL6, 预后, Diffuse large B-cell lymphoma, BCL2, P53, BCL6, Prognosis

## Abstract

**目的:**

筛选并分析与弥漫大B细胞淋巴瘤（DLBCL）预后相关的免疫表型，探究其预后价值。

**方法:**

选取天津医科大学肿瘤医院2011年1月至2016年12月收治的163例DLBCL患者，免疫组织化学染色检测DLBCL常见免疫表型，COX模型探索独立于国际预后指数（IPI）影响总生存（OS）与无进展生存（PFS）的免疫表型，并分析其两两联合表达对预后的影响。

**结果:**

多因素分析显示BCL6阴性（PFS：*HR*＝1.652，95％ *CI* 1.030～2.649，*P*＝0.037）、P53阳性（OS：*HR*＝1.842，95％ *CI* 1.008～3.367，*P*＝0.047）、BCL2强阳性（OS：*HR*＝2.102，95％*CI* 1.249～3.537，*P*＝0.005；PFS：*HR*＝2.126，95％ *CI* 1.312～3.443，*P*＝0.002）是DLBCL中独立于IPI的预后不良因素。亚组分析显示，在年龄≤60岁组患者中BCL6阴性（PFS：*HR*＝2.042，95％*CI* 1.021～4.081，*P*＝0.043）、P53阳性（OS：*HR*＝3.069，95％ *CI* 1.244～7.569，*P*＝0.015）和BCL2强阳性（OS：*HR*＝2.433，95％ *CI* 1.165～5.082，*P*＝0.018；PFS：*HR*＝3.209，95％*CI* 1.606～6.410，*P*＝0.001）对预后影响显著；在IPI 0～2分亚组患者中，BCL6阴性（OS：*HR*＝2.467，95％*CI* 1.322～4.604，*P*＝0.005；PFS：*HR*＝2.248，95％*CI* 1.275～3.965，*P*＝0.005）和BCL2强阳性（PFS：*HR*＝2.045，95％*CI* 1.119～3.735，*P*＝0.020）对预后影响显著。BCL6和BCL2强阳性的联合表达与DLBCL的预后相关（*P*＝0.005和*P*<0.001），BCL6阳性/BCL2非强阳性（86例）预后最好［3年OS率（71.6±4.9）％，3年PFS率（67.0±5.1）％］，BCL6阴性/BCL2强阳性（10例）预后最差［3年OS率（20.0±12.6）％，3年PFS率（10.0±9.5）％］；BCL6、P53的联合表达与DLBCL的预后差异无统计学意义（*P*＝0.061和*P*＝0.089），但生存曲线显示BCL6阳性/P53阴性的病例（98例）预后较好［3年OS率（70.6±4.7）％，3年PFS率（64.6±4.9）％］；BCL2强阳性、P53的联合表达与DLBCL的预后显著相关（*P*<0.001和*P*<0.001），BCL2强阳性/P53阳性的病例（5例）预后最差（3年OS率和PFS率均为0）；无论BCL6与P53表达如何，BCL2强阳性的病例预后均比非强阳性病例差。

**结论:**

BCL6阴性、P53阳性、BCL2强阳性三种免疫表型单独及联合表达对DLBCL尤其是年龄≤60岁和IPI 0～2分患者的预后预测具有一定价值。

## Abstract (en)

**Objective:**

To screen and analyze the prognostic protein biomarkers of DLBCL, and to explore their value in the prognostic evaluation.

**Methods:**

163 cases of confirmed DLBCLs from January 2011 to December 2016 were collected with their clinical, pathological and follow-up data, which were all from our hospital. The expression of protein markers were tested using immunohistochemical staining（IHC）. The immune phenotypes independent of the International Prognostic Index（IPI）that affect overall survival（OS）and progression-free survival（PFS）of DLBCL were explored by COX regression model, and the effect of their co-expression on the prognosis were also analyzed.

**Results:**

BCL6 negative（PFS: *HR*＝1.652, 95％ *CI* 1.030–2.649, *P*＝0.037）, P53 positive（OS: *HR*＝1.842, 95％ *CI* 1.008–3.367, *P*＝0.047）, and BCL2 strong positive expressions（S +）（OS: *HR*＝2.102, 95％ *CI* 1.249–3.537, *P*＝0.005; PFS: *HR*＝2.126, 95％ *CI* 1.312–3.443, *P*＝0.002）are adverse prognostic factors of DLBCL that are independent of IPI. BCL6^−^（PFS: *HR*＝2.042, 95％ *CI* 1.021–4.081, *P*＝0.043）, P53 ^+^（OS: *HR*＝3.069, 95％*CI* 1.244–7.569, *P*＝0.015）and BCL2^S+^（OS: *HR*＝2.433, 95％*CI* 1.165–5.082, *P*＝0.018; PFS: *HR*＝3.209, 95％*CI* 1.606–6.410, *P*＝0.001）are adverse prognostic factors in the group of age≤60-year-old; in the group of IPI score 0–2, cases with BCL6^−^（OS: *HR*＝2.467, 95％*CI* 1.322–4.604, *P*＝0.005; PFS: *HR*＝2.248, 95％*CI* 1.275–3.965, *P*＝0.005）and BCL2^S +^（PFS: *HR*＝2.045, 95％*CI* 1.119–3.735, *P*＝0.020）have worse prognosis. The co-expression of BCL6^−^ and BCL2^S +^ has significant influence on prognosis of DLBCL（*P*＝0.005 and *P*<0.001）, in which BCL6^+^/non-BCL2^S+^（*n*＝86）has the best prognosis［3-year-OS（71.6±4.9）％, 3-year-PFS（67.0±5.1）％］, and BCL6^−^/BCL2^S+^（*n*＝10）has the worst prognosis［3-year-OS（20.0±12.6）％, 3-year-PFS（10.0±9.5）％］; the co-expression of BCL6^−^ and P53^+^ has no significant influence on prognosis（*P*＝0.061 and *P*＝0.089）, however, those cases with BCL6^+^/P53^−^（*n*＝98）often get better prognosis［3-year-OS（70.6±4.7）％, 3-year-PFS（64.6±4.9）％］than others; the co-expression of P53^+^ and BCL2^S +^ has significant influence on prognosis of DLBCL（*P*<0.001 and *P*<0.001）, and P53^+^/BCL2^S+^（*n*＝5）has the worst prognosis（3-year-OS and 3-year-PFS are both 0）; BCL2^S+^ cases get shorter OS and PFS, regardless of the expression of BCL6 and P53.

**Conclusion:**

The expression and coexpression of BCL6 negative, P53 positive and BCL2^S +^ have certain value in the prognostic evaluation of DLBCL, especially in the group of age≤60-year-old and IPI score 0–2.

弥漫大B细胞淋巴瘤（DLBCL）是成年人群中最常见的非霍奇金淋巴瘤（NHL）[1]，在R-CHOP（利妥昔单抗+环磷酰胺+长春新碱+阿霉素+泼尼松）靶向联合化疗条件下，仍有30％～40％的病例预后较差，表现为初治后短期复发或耐药难治[2]。影响DLBCL预后的相关因素很多，自1993年以来，国际预后指数（IPI）是DLBCL中应用最广泛的临床预后分层模型[3]，多年来在DLBCL尤其是IPI评分为0～2分的低危组病例中具有很好的临床指导作用[4]，随着研究和认识的进步，越来越多独立于IPI的预后因素被发现，包括血液生化指标以及肿瘤组织的免疫组化指标等。在病理研究中，很多蛋白的表达与DLBCL预后有一定关联性，如CD10[5]、MUM1[6]、BCL6[7]、BCL2阳性及强阳性[4],[8]、MYC[9]、P53[10]–[11]、Ki67[12]等。本研究旨在分析这些DLBCL中常见免疫表型的预后意义，探究DLBCL独立于IPI的预后相关免疫表型。

## 病例与方法

一、研究对象

以我院2011年1月至2016年12月术后经组织病理确诊并接受3个周期及以上CHOP或R-CHOP治疗的163例DLBCL患者为研究对象，临床和影像学资料要求完整，采集信息包括年龄、部位、COO分型、Ann Arbor分期、LDH、β_2_微球蛋白等。选取合适的蜡块进行组织芯片制作，经FISH检测排除“双打击”及“三打击”淋巴瘤后，应用免疫组织化学方法检测CD10、MUM1、BCL6、BCL2、MYC、P53、Ki67的表达。

163例DLBCL病例随访截至2019年6月，中位随访时间为73（31～99）个月。无进展生存（PFS）时间定义为从患者诊断至复发、死亡或随访截止时间，总生存（OS）时间为从诊断至死亡或随访截止时间。

二、免疫组织化学染色结果判读

包括CD10、MUM1、BCL6、BCL2、MYC、P53、Ki67抗体均购自北京中杉金桥生物技术公司，并按照说明书进行免疫组织化学染色操作。结果由专科病理医师及专家判读并审核，将染色结果中的淡黄、黄和棕黄分别判定为+、++和+++，通过计算肿瘤细胞着色百分比来确定表达情况。其中CD10、BCL6、MUM1以任何染色强度且肿瘤细胞≥30％阳性为阳性[13]；Ki67以中度以上染色（++、+++）且肿瘤细胞≥80％阳性为高表达；MYC以中度以上染色且肿瘤细胞≥40％阳性为阳性[14]，P53以中度以上染色且肿瘤细胞≥50％阳性为阳性[15]；BCL2以中度以上染色且肿瘤细胞≥50％阳性为阳性[14]；BCL2强染色（+++）且肿瘤细胞≥90％阳性判定为强阳性[8]。同时出现MYC≥40％阳性和BCL2≥50％阳性的病例为双表达淋巴瘤（DEL）[16]。

三、统计学处理

采用SPSS 17.0进行统计学分析。分类变量的组间比较采用卡方检验或Fisher精确概率法；预后的单因素和多因素分析采用Cox比例风险模型；采用Kaplan-Meier绘制生存曲线，组间比较采用Log-rank检验。*P*<0.05为差异有统计学意义。

## 结果

一、一般临床特征

163例DLBCL一般临床特征见表1。经Hans分型，73例为生发中心B细胞样（GCB）亚型（44.8％），90例为non-GCB亚型（55.2％）；结内淋巴瘤84例，占51.5％；年龄≤60岁86例，占52.8％；Ann Arbor Ⅰ/Ⅱ期94例，占57.7％；IPI 0～2分的病例119例，占73.0％；血浆LDH水平升高的病例62例，占38.0％。

**表1:** 163例弥漫大B细胞淋巴瘤患者一般临床特征

临床特征	例数（％）
性别	
男	89（54.6）
女	74（45.4）
年龄	
≤60岁	86（52.8）
>60岁	77（47.2）
受累部位	
结内	84（51.5）
结外	79（48.5）
COO分型	
GCB	73（44.8）
non-GCB	90（55.2）
Ann Arbor分期	
Ⅰ/Ⅱ期	94（57.7）
Ⅲ/Ⅳ期	69（42.3）
IPI评分	
0～2分	119（73.0）
3～5分	44（27.0）
LDH	
≤247U/L	101（62.0）
>247U/L	62（38.0）

注：GCB：生发中心B细胞样亚型；non-GCB：非生发中心B细胞样亚型；IPI：国际预后指数

二、预后相关免疫表型的筛选

1. 单因素和多因素分析：单因素及多因素Cox回归结果见表2～3，将单因素分析中*P*<0.05的变量纳入多因素分析。结果显示，P53阳性（*P*＝0.047）、BCL2强阳性（*P*＝0.005）和IPI 3～5分（*P*＝0.018）是影响OS的独立危险因素，而BCL2强阳性（*P*＝0.002）、BCL6阴性（*P*＝0.037）和IPI 3～5分（*P*＝0.008）是影响PFS的独立危险因素。综合OS和PFS结果，独立于IPI的DLBCL预后相关免疫表型包括BCL2强阳性、P53阳性和BCL6阴性（图1）。

**表2:** 影响弥漫大B细胞淋巴瘤患者总生存的单因素和多因素分析

影响因素	单因素分析		多因素分析	
	*HR*（95％ *CI*）	*P*值	*HR*（95％ *CI*）	*P*值
结内受累	1.101（0.677～1.792）	0.697		
DEL	1.017（0.585～1.770）	0.951		
non-GCB	1.046（0.639～1.710）	0.859		
BCL2阳性	1.341（0.791～2.272）	0.276		
BCL2强阳性	2.020（1.216～3.354）	0.007	2.102（1.249～3.537）	0.005
MYC阳性	0.722（0.423～1.233）	0.233		
P53阳性	1.865（1.046～3.323）	0.035	1.842（1.008～3.367）	0.047
BCL6阴性	1.508（0.901～2.522）	0.118		
CD10阳性	1.461（0.847～2.519）	0.173		
Ki67阳性	0.895（0.550～1.458）	0.657		
MUM1阴性	0.787（0.479～1.293）	0.344		
β_2_-MG>2.7mg/L	1.057（0.640～1.746）	0.829		
IPI3～5分	2.161（1.302～3.587）	0.003	1.877（1.114～3.162）	0.018

注：DEL：双表达淋巴瘤；non-GCB：非生发中心亚型；IPI：国际预后指数；β_2_-MG：β_2_微球蛋白

**表3:** 影响弥漫大B细胞淋巴瘤患者无进展生存的单因素和多因素分析

影响因素	单因素分析		多因素分析	
	*HR*（95％ *CI*）	*P*值	*HR*（95%*CI*）	*P*值
结内受累	0.893（0.571～1.396）	0.619		
DEL	1.287（0.791～2.095）	0.310		
non-GCB	1.072（0.683～1.682）	0.763		
BCL2阳性	1.448（0.894～2.346）	0.132		
BCL2强阳性	2.041（1.272～3.275）	0.003	2.126（1.312～3.443）	0.002
MYC阳性	0.894（0.558～1.434）	0.642		
P53阳性	1.544（0.891～2.677）	0.121		
BCL6阴性	1.600（1.007～2.542）	0.047	1.652（1.030～2.649）	0.037
CD10阳性	1.280（0.768～2.132）	0.343		
Ki67阳性	0.867（0.555～1.356）	0.532		
MUM1阴性	0.859（0.548～1.347）	0.509		
β_2_-MG>2.7mg/L	1.281（0.816～2.009）	0.281		
IPI3～5分	2.036（1.279～3.239）	0.003	1.873（1.175～2.986）	0.008

注：DEL：双表达淋巴瘤；non-GCB：非生发中心亚型；IPI：国际预后指数；β_2_-MG：β_2_微球蛋白

**图1:**
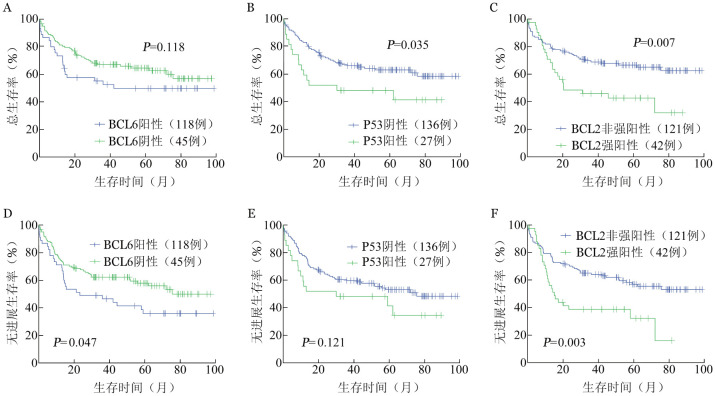
不同BCL6、P53和BCL2表达分组弥漫大B细胞淋巴瘤患者总生存（A～C）与无进展生存（D～F）曲线 A、D：BCL6表达；B、E：P53表达；C、F：BCL2表达

2. BCL6阴性、P53阳性、BCL2强阳性的分布：BCL6阴性、P53阳性和BCL2强阳性在DLBCL患者各临床特征中的分布见表4。non-GCB亚型中BCL6阴性病例多见（93.3％对40.7％，*P*<0.001）；IPI 3～5分患者组中P53阳性的病例多见（44.4％对23.5％，*P*＝0.046）；在结内受累（71.4％对44.6％，*P*＝0.005）和Ann Arbor Ⅲ/Ⅳ期（57.1％对37.2％，*P*＝0.038）患者中，BCL2强阳性的构成比更高。提示三种免疫表型在DLBCL病例中的分布具有一定差异。

**表4:** BCL6阴性、P53阳性和BCL2强阳性在弥漫大B细胞淋巴瘤患者各临床特征中的分布

临床特征	BCL6			P53			BCL2		
	阴性（45例）	阳性（118例）	*P*值	阳性（27例）	阴性（136例）	*P*值	强阳性（42例）	非强阳性（121例）	*P*值
年龄			0.663			0.114			0.631
≤60岁	22（48.9）	64（54.2）		10（37.0）	76（55.9）		24（57.1）	62（51.2）	
>60岁	23（51.1）	54（45.8）		17（63.0）	60（44.1）		18（42.9）	59（48.8）	
COO分型			<0.001			0.802			0.543
GCB	3（6.7）	70（59.3）		11（40.7）	62（45.6）		21（50.0）	52（43.0）	
non-GCB	42（93.3）	48（40.7）		16（59.3）	74（54.4）		21（50.0）	69（57.0）	
受累部位			0.418			0.150			0.005
结内	26（57.8）	58（49.2）		10（37.0）	74（54.4）		30（71.4）	54（44.6）	
结外	19（42.2）	60（50.8）		17（63.0）	62（45.6）		12（28.6）	67（55.4）	
IPI评分			0.889			0.046			0.202
0～2分	32（71.1）	87（73.7）		15（55.6）	104（76.5）		27（64.3）	92（76.0）	
3～5分	13（28.9）	31（26.3）		12（44.4）	32（23.5）		15（35.7）	29（24.0）	
Ann Arbor分期			0.385			0.976			0.038
Ⅰ/Ⅱ期	23（51.1）	71（60.2）		15（55.6）	79（58.1）		18（42.9）	76（62.8）	
Ⅲ/Ⅳ期	22（48.9）	47（39.8）		12（44.4）	57（41.9）		24（57.1）	45（37.2）	
LDH			0.999			0.161			0.352
≤247U/L	28（62.2）	73（61.9）		13（48.1）	88（64.7）		23（54.8）	78（64.5）	
>247U/L	17（37.8）	45（38.1）		14（51.9）	48（35.3）		19（45.2）	43（35.5）	

注：GCB：生发中心B细胞样亚型；non-GCB：非生发中心B细胞样亚型；IPI：国际预后指数

三、亚组分析

进一步按照IPI评分（0～2分，3～5分）、年龄（≤60岁，>60岁）、LDH水平（≤247 U/L，>247 U/L）和Ann Arbor分期（Ⅰ/Ⅱ期，Ⅲ/Ⅳ期）进行分组，分析不同亚组中BCL6阴性、P53阳性和BCL2强阳性表达的预后意义，结果见表5～6。

**表5:** 不同亚组中BCL6阴性、P53阳性和BCL2强阳性对总生存影响的单因素分析

组别	BCL6阴性		P53阳性		BCL2强阳性	
	*HR*（95％*CI*）	*P*值	*HR*（95％*CI*）	*P*值	*HR*（95％*CI*）	*P*值
年龄						
≤60岁	2.102（0.991～4.456）	0.053	3.069（1.244～7.569）	0.015	2.433（1.165～5.082）	0.018
>60岁	1.103（0.542～2.244）	0.788	1.214（0.571～2.582）	0.615	1.824（0.892～3.730）	0.100
LDH水平						
≤247U/L	1.812（0.911～3.606）	0.090	0.621（0.190～2.030）	0.430	2.057（1.018～4.155）	0.044
>247U/L	1.189（0.544～2.599）	0.664	3.294（1.570～6.913）	0.002	1.869（0.894～3.908）	0.097
AnnArbor分期						
Ⅰ/Ⅱ期	3.121（1.511～6.444）	0.002	1.785（0.764～4.166）	0.181	1.544（0.657～3.627）	0.319
Ⅲ/Ⅳ期	0.700（0.327～1.496）	0.357	1.952（0.885～4.306）	0.098	1.983（1.014～3.876）	0.045
IPI评分						
0～2分	2.467（1.322～4.604）	0.005	1.698（0.752～3.833）	0.203	1.801（0.930～3.489）	0.081
3～5分	0.521（0.194～1.401）	0.196	1.521（0.650～3.559）	0.333	2.044（0.904～4.621）	0.086

注：LDH：乳酸脱氢酶；IPI：国际预后指数

**表6:** 不同亚组中BCL6阴性、P53阳性和BCL2强阳性对无进展生存影响的单因素分析

组别	BCL6阴性		P53阳性		BCL2强阳性	
	*HR*（95％*CI*）	*P*值	*HR*（95％*CI*）	*P*值	*HR*（95％*CI*）	*P*值
年龄						
≤60岁	2.042（1.021～4.081）	0.043	2.337（0.965～5.660）	0.060	3.209（1.606～6.410）	0.001
>60岁	1.245（0.666～2.326）	0.493	1.024（0.506～2.074）	0.948	1.399（0.704～2.779）	0.337
LDH水平						
≤247U/L	1.876（1.021～3.447）	0.043	0.628（0.224～1.758）	0.376	2.353（1.247～4.442）	0.008
>247U/L	1.311（0.638～2.694）	0.461	2.896（1.411～5.943）	0.004	1.622（0.796～3.308）	0.183
Ann Arbor分期						
Ⅰ/Ⅱ期	2.801（1.412～5.558）	0.003	1.495（0.649～3.439）	0.345	1.314（0.569～3.034）	0.523
Ⅲ/Ⅳ期	0.840（0.444～1.588）	0.591	1.649（0.791～3.438）	0.182	2.168（1.175～4.000）	0.013
IPI评分						
0～2分	2.248（1.275～3.965）	0.005	1.281（0.576～2.850）	0.543	2.045（1.119～3.735）	0.020
3～5分	0.747（0.328～1.702）	0.488	1.412（0.638～3.125）	0.394	1.757（0.810～3.814）	0.154

注：LDH：乳酸脱氢酶；IPI：国际预后指数

年龄≤60岁组中，P53阳性（*P*＝0.015）、BCL2强阳性（*P*＝0.018）是影响OS的危险因素，BCL6阴性（*P*＝0.043）、BCL2强阳性（*P*＝0.001）是影响PFS的危险因素；年龄>60岁患者中BCL6阴性、P53阳性和BCL2强阳性对预后无明显影响。LDH≤247 U/L亚组中，BCL2强阳性同时影响OS和PFS（*P*值分别为0.044、0.008），BCL6阴性影响PFS（*P*＝0.043）；LDH>247 U/L亚组中，P53是影响OS及PFS的危险因素（*P*值分别为0.002、0.004）。Ann Arbor Ⅰ/Ⅱ期组中，BCL6阴性是影响OS和PFS的危险因素（*P*值分别为0.002、0.003）；Ann Arbor Ⅲ/Ⅳ期组中，BCL2强阳性影响OS与PFS（*P*值分别为0.045、0.013）。IPI 0～2分亚组中，BCL6阴性影响OS和PFS（*P*值均为0.005），BCL2强阳性影响PFS（*P*＝0.020）；IPI 3～5分亚组中，BCL6阴性、P53阳性和BCL2强阳性对预后无明显影响。

可以看出，在年龄≤60岁组，BCL6阴性、P53阳性和BCL2强阳性对预后的影响较为显著；IPI 0～2分亚组中，BCL6阴性和BCL2强阳性对预后的影响较为显著。提示低年龄分组与IPI低分组受免疫组化的影响更大。

四、BCL6阴性、P53阳性、BCL2强阳性两两联合表达对预后的影响

BCL6阴性、P53阳性及BCL2强阳性两两联合表达的预后分析见图2。

**图2:**
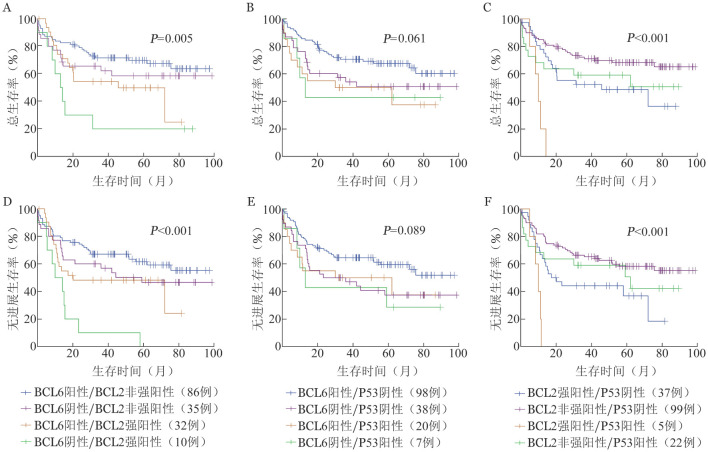
不同BCL6、P53和BCL2两两联合表达弥漫大B细胞淋巴瘤患者总生存（A～C）与无进展生存（D～F）曲线 A、D：BCL6、BCL2联合表达；B、E：BCL6、P53联合表达；C、F：BCL2、P53联合表达

BCL6阳性/BCL2非强阳性（86例）、BCL6阴性/BCL2非强阳性（35例）、BCL6阳性/BCL2强阳性（32例）、BCL6阴性/BCL2强阳性（10例）组3年OS率分别为（71.6±4.9）％、（62.1±8.3）％、（54.3±9.0）％、（20.0±12.6）％（*P*＝0.005），3年PFS率分别为（67.0±5.1）％、（46.6±8.8）％、（48.2±9.0）％、（10.0±9.5）％（*P*<0.001）。BCL6阳性/BCL2非强阳性预后最好，BCL6阴性/BCL2强阳性预后最差；且无论BCL6表达如何，BCL2强阳性组预后更差。

BCL6阳性/P53阴性（98例）、BCL6阴性/P53阴性（38例）、BCL6阳性/P53阳性（20例）、BCL6阴性/P53阳性（7例）组3年OS率分别为（70.6±4.7）％、（50.8±8.4）％、（50.0±11.2）％、（42.9±18.7）％（*P*＝0.061），3年PFS率分别为（64.6±4.9）％、（37.4±8.2）％、（50.0±11.2）％、（42.9±18.7）％（*P*＝0.089）。BCL6、P53的联合表达与DLBCL的预后差异无统计学意义，BCL6阳性/P53阴性患者预后最好。

BCL2非强阳性/P53阴性（99例）、BCL2强阳性/P53阴性（37例）、BCL2非强阳性/P53阳性（22例）、BCL2强阳性/P53阳性（5例）组3年OS率分别为（73.3±4.5）％、（52.3±8.4）％、（59.1±10.5）％、0（*P*<0.001），3年PFS率分别为（65.2±4.8）％、（44.2±8.3）％、（59.1±10.5）％、0（*P*<0.001）。BCL2非强阳性/P53阴性的病例预后最好；BCL2强阳性/P53阳性病例预后最差；无论P53表达如何，BCL2强阳性患者的预后均较BCL2非强阳性患者差。

## 讨论

影响DLBCL预后的因素很多[17]，可分为：①临床特征指标，包括IPI、B症状、结外受累器官部位及数量、体重指数（BMI）、年龄等；②血液中的生化指标，包括淋巴细胞和单核细胞计数、维生素D水平、游离DNA（ct-DNA）含量、IL-2受体等；③影像和放射学检查结果，如PET-CT评估下的肿瘤体积和肿瘤代谢活性等；④肿瘤细胞免疫表型：如P53、BCL2、BCL6、MYC等蛋白的表达，以及CD5、CD20、CD30、CD37、FOXP1、Ki67等蛋白标志物的表达；⑤基因和染色体等遗传学异常：如COO分型，MYC、BCL2、BCL6、IGH等基因重排，P53、MYD88、CD79B等基因突变。其中，基于免疫组织化学染色对肿瘤细胞表达的特定蛋白分子的检测是病理诊断中一项重要的检查项目，有助于肿瘤的诊断和鉴别诊断；同时很多蛋白的表达也可预示肿瘤的良恶性、侵袭性，进一步对治疗反应、预后进行预测。免疫组化检测相对于基因和染色体检测更加直观而且操作简单，在基因突变的初筛中具有较实用的价值。

IPI评分由年龄、ECOG评分、Ann Arbor分期、LDH水平和结外累及状况构成。但近年来的研究显示，IPI在预测接受利妥昔单抗治疗患者的OS时具有差异性较大而一致性较低，而且对IPI评分为3～5分的高危组病例的预后分层能力较差[18]–[19]。研究人员也提出一系列衍生的IPI模型，如年龄调整后的IPI（aaIPI）、改良IPI（R-IPI）、美国国家综合癌症网络IPI（NCCN-IPI）[18]，以及针对某一特定部位如中枢神经系统IPI（CNS-IPI）等。这些不同的IPI模型均采用了相似的评分指标，在不同的层次上进行了细化分类。已有研究表明NCCN-IPI对高危组病例的预测和分层能力要好于IPI。除IPI包含的几项评分指标外，一些独立于IPI的预后影响因素也在不断被提出，有研究发现将红细胞容积分布密度（RDW）和PLT两指标与IPI结合后，对R-CHOP治疗下的DLBCL患者的预后分层能力较IPI更强[20]。

我们通过对排除双打击和三打击后的163例DLBCL患者进行预后分析，筛选出BCL6阴性、P53阳性和BCL2强阳性作为独立于IPI的预后相关免疫组化表型，并对三者单独和联合表达对DLBCL的预后影响进行了探究，结果显示三者尤其在低年龄组、LDH正常组、低IPI评分的低危组病例中对预后的影响较明显。在这一研究基础上，能否将预后相关免疫表型单一或组合地与IPI结合构建出对特定DLBCL病例具有更好预测作用的预后评价模型，对于当下将病理与临床结合对DLBCL进行进一步分层诊疗具有指导意义。

BCL6是位于染色体3q27的编码抑制因子的基因，具有多种抑制和负向调节作用，如阻止细胞周期阻滞、抑制BCL2的抗凋亡作用、防止终末分化等。BCL6蛋白是作为Hans分型的一个常规检测的组化指标，尽管BCL6蛋白经常作为与MYC或BCL2共表达被研究，但单独BCL6表达与DLBCL的预后关系尚存在争议。有研究显示BCL6>25％+的DLBCL患者预后要好于BCL6阴性的患者，且BCL6阳性是独立于IPI的预后影响因素[21]，但仍需进一步探究和证实。本研究显示，BCL6阴性为DLBCL的独立预后不良因素，虽然相对于P53阳性和BCL2强阳来说对预后的影响相对较弱，但在其与BCL2强阳及P53阳性联合时，患者较单一的P53阳性或BCL2强阳表达病例具有更短的3年OS和PFS率。

P53基因突变会使其对细胞周期的调节功能丧失，使细胞异常增殖及发生恶性转化，P53基因突变也是肿瘤基因检测的常规检查项目之一。有研究表明，当判定P53蛋白高表达cut-off＝50％时，用P53蛋白表达检测替代TP53基因突变检测具有较高的特异度，P53蛋白表达>50％+可用于在TP53测序不便时给DLBCL患者进行诊断分层[22]。而进一步研究发现，P53蛋白高表达对P53基因突变的预测敏感度为55.6％，特异度为90.8％，P53蛋白高表达与TP53基因突变的一致率为88.8％。故通过P53蛋白的表达情况可对P53基因的突变做初步筛查[11]，结合其对预后的影响，P53蛋白高表达对于DLBCL的诊疗和预后预测均具有更广泛的研究和应用价值。

BCL2是位于染色体18q21的一个具有抗细胞凋亡作用的癌基因，广泛表达于未成熟B细胞和记忆B细胞中。在DLBCL中，约有50％的病例存在BCL2的高表达（≥50％+）[4]。BCL2的高表达和过表达对预后的影响目前尚存在争议，但已有研究证实，BCL2强阳性的病例OS和PFS均较差，而且BCL2强阳性是独立于IPI和MYC表达的预后影响因素[8]。在本研究中，BCL2强阳性是DLBCL中的较为显著的预后不良因素，单独或联合其他因素的BCL2强阳性患者均具有最差的OS和PFS。BCL2与MYC蛋白双表达被认为预后较差，但本组中，按照WHO定义的[1]双表达病例与非双表达病例在预后上差异并无统计学意义；但在本研究DEL病例组中，P53阳性与BCL2强阳性联合表达的病例预后明显较差（*P*＝0.014），与文献[4],[23]结果一致。
